# The Effects of Age, Adiposity, and Physical Activity on the Risk of Seven Site‐Specific Fractures in Postmenopausal Women

**DOI:** 10.1002/jbmr.2826

**Published:** 2016-05-05

**Authors:** Jason Lacombe, Benjamin J Cairns, Jane Green, Gillian K Reeves, Valerie Beral, Miranda EG Armstrong, for the Million Women Study collaborators

**Affiliations:** ^1^ Faculty of Kinesiology & Physical EducationUniversity of TorontoTorontoCanada; ^2^ Cancer Epidemiology UnitUniversity of OxfordOxfordUnited Kingdom

**Keywords:** FRACTURE PREVENTION, EXERCISE, GENERAL POPULATION STUDIES, EPIDEMIOLOGY, OSTEOPOROSIS

## Abstract

Risk factors for fracture of the neck of the femur are relatively well established, but those for fracture at other sites are little studied. In this large population study we explore the role of age, body mass index (BMI), and physical activity on the risk of fracture at seven sites in postmenopausal women. As part of the Million Women Study, 1,154,821 postmenopausal UK women with a mean age of 56.0 (SD 4.8) years provided health and lifestyle data at recruitment in 1996 to 2001. All participants were linked to National Health Service (NHS) hospital records for day‐case or overnight admissions with a mean follow‐up of 11 years per woman. Adjusted absolute and relative risks for seven site‐specific incident fractures were calculated using Cox regression models. During follow‐up, 4931 women had a fracture of the humerus; 2926 of the forearm; 15,883 of the wrist; 9887 of the neck of the femur; 1166 of the femur (not neck); 3199 a lower leg fracture; and 10,092 an ankle fracture. Age‐specific incidence rates increased gradually with age for fractures of forearm, lower leg, ankle, and femur (not neck), and steeply with age for fractures of neck of femur, wrist, and humerus. When compared to women with desirable BMI (20.0 to 24.9 kg/m^2^), higher BMI was associated with a reduced risk of fracture of the neck of femur, forearm, and wrist, but an increased risk of humerus, femur (not neck), lower leg, and ankle fractures (*p* < 0.001 for all). Strenuous activity was significantly associated with a decreased risk of fracture of the humerus and femur (both neck and remainder of femur) (*p* < 0.001), but was not significantly associated with lower leg, ankle, wrist, and forearm fractures. Postmenopausal women are at a high lifetime risk of fracture. BMI and physical activity are modifiable risk factors for fracture, but their associations with fracture risk differ substantially across fracture sites. © 2016 The Authors. *Journal of Bone and Mineral Research* published by Wiley Periodicals, Inc. on behalf of American Society for Bone and Mineral Research (ASBMR)

## Introduction

For postmenopausal women, fractures are a major cause of morbidity and mortality,^1^ and preventive behaviors may include regular physical activity and managing adiposity. Although many studies have reported that increased physical activity is associated with a reduced risk of fracture of the neck of the femur (hip),^2^, ^3^, ^4^, ^5^, ^6^ its association with fracture risk at different sites remains understudied and may be complex. Body mass index (BMI) is a strong determinant of bone mineral density and whereas previous research has shown that obesity is protective against fracture at the neck of the femur,^2^, ^3^, ^7^, ^8^, ^9^ associations at other fracture sites remain poorly understood.

In this large, population‐based, prospective cohort study of postmenopausal women, we compare the relationship of age, BMI, and physical activity with the risk of fractures at seven sites: humerus, forearm, wrist, neck of the femur, femur (not neck), lower leg, and ankle. In a previous study using Million Women Study data we reported varying associations of BMI and physical activity with fractures of the hip, wrist, and ankle.^2^ With the current analyses we update the earlier findings with an additional 3 years of follow‐up, and include new results from four additional fracture sites.

## Materials and Methods

### Participants and data

The Million Women Study is a population‐based, prospective cohort study of women in the United Kingdom. Details of study design and methods have been described elsewhere.^10^ Briefly, 1.3 million women were invited for breast screening at various National Health Service (NHS) clinics in England and Scotland. Participants were recruited into the study in 1996 to 2001 through completing a questionnaire that included questions on anthropometry, physical activity, and other lifestyle, demographic, and medical information. Written consent to participate was obtained from each participant, and the Oxford and Anglia Multi‐Centre Research Ethics Committee provided ethics approval. Study questionnaires and further details of the data and access policies can be viewed on the website (www.millionwomenstudy.org).

Each woman's unique NHS identification number, as well as other personal information, was used to link to cause‐specific information on NHS hospital admission databases: Hospital Episodes Statistics for England,^11^ and Scottish Morbidity Records in Scotland.^12^ These databases include information on inpatient (ie, overnight) stays and day‐case admissions (ie, women admitted and discharged on the same day).

Women's hospital admission records included the date of admission and were coded using the World Health Organization's International Classification of Diseases 10^th^ revision (ICD‐10) for diagnoses.^13^ Incident cases of fracture were defined as the first hospital record (day or overnight admissions) of fracture (either primary or secondary diagnosis) of the humerus (S42.2 to S42.4); forearm (S52.0 to S52.4, S52.7); wrist (S52.5 to S52.6, S62.0 to S62.1, S62.8); neck of the femur (S72.0 to S72.2); femur (not neck) (S72.3 to S72.4); lower leg (S82.1 to S82.2, S82.4); or ankle (S82.3, S82.5 to S82.6, S82.8) after recruitment into the study. Because each fracture analysis was run separately, if multiple fractures occurred simultaneously for any of the main fracture sites, the fracture was assigned to the current fracture of interest for the individual analysis. Because prior fracture is a risk factor for subsequent fracture,^14^ we censored at the first occurrence of any fracture (see below). All other fractures were defined as ICD‐10 codes: M48.4, M80.0, M84.3, S02, S12, S22, S32, S42, S52, S62, S72, S82, S92, T02, T08, T10, T12, T14.2.

### Measure of body size, physical activity, and other factors

At recruitment, women reported their height in feet and inches, and their weight in stones and pounds. This was used to calculate BMI as weight (kg)/height (m)^2^. To assess the combined effects of measurement error and changes in BMI over the follow‐up period, a sample of 3501 women in the study had their weight and height measured by their general practitioners 9 years after reporting of height and weight at recruitment.^15^ We found excellent agreement (correlation coefficient = 0.85) between self‐reported baseline BMI and measured BMI 9 years later. The regression dilution ratio for BMI was 0.98, indicating that reporting errors in BMI are likely to have only a very small effect on estimates of risk associations in log‐linear models.

To assess physical activity, frequency of strenuous activity was assessed by asking, “How often do you do any strenuous exercise (ie, enough to cause sweating or a fast heartbeat)?” And total frequency of any activity was assessed by the question, “How often do you do any exercise?” Each question had the options: rarely/never, less than once a week, once a week, two to three times a week, four to six times a week, every day. The first 9% of the recruitment questionnaires did not ask the questions on any physical activity. Self‐reported total number of hours doing strenuous activity, cycling, and occupational activity is correlated with objective measures of physical activity.^16^ We previously assessed the ability of the baseline questions to discriminate between hours of reported time spent walking, gardening, cycling, and doing strenuous activity; we did not include occupational activity because only 20% of the women reported being in full‐time paid work at the time.^17^

### Analysis

All data were analyzed using the statistical package Stata, version 13.1^18^ (Stata Corporation, Inc., College Station, TX, USA). Person‐years were calculated from the date of recruitment. For women in England, recording of hospital data was incomplete before April 1, 1997, and for the 5% of women recruited before then, follow‐up was calculated from that date. For Scotland, hospital data were available from January 1, 1981. Follow‐up was censored at whichever of the following came first: the date of any fracture (see above); date of death; date of emigration; or the end of follow‐up. For participants in England, the last date of follow‐up was March 31, 2011; for Scotland, this date was December 31, 2008.

Menopausal status of women at recruitment was defined by their reported menstrual history. Those who reported at recruitment that they had experienced natural menopause (49%), or had undergone bilateral oophorectomy (6%) were defined as postmenopausal. Women who were premenopausal, perimenopausal, or of unknown menopausal status at baseline, were assumed to be postmenopausal after they reached the age of 55 years because 96% of women in this cohort with a known age of natural menopause were postmenopausal by that age.

Women were excluded if they had a hospital record of fracture or a diagnosis of cancer before recruitment, and if at recruitment they reported being treated for osteoporosis or having had a stroke. These exclusions were applied because these conditions might affect subsequent weight, physical activity, bone mineral density, and the likelihood of falls.^14^, ^19^

Cox regression models were used to calculate relative risks (RRs) and 95% confidence intervals (CIs) for fracture at each site studied in relation to BMI and to physical activity, with attained age as the underlying time variable. Analyses were stratified by recruitment region (10 regions), and adjusted for: parity (nulliparous or parous); height (<155, 155.0 to 159.9, 160 to 164.9, 165.0 to 169.9, or ≥170 cm); smoking status (current, past, never); alcohol consumption (0, 0.1 to 2.9, 3 to 6.9, 7 to 14.9, ≥15 units per week, 1 unit = 10 g); socioeconomic status (quintiles using the Townsend index)^20^; history of heart disease/thrombosis (yes, no); history of osteoarthritis/rheumatoid arthritis (yes, no); history of thyroid disease (yes, no); and menopausal hormone‐replacement therapy (HRT) use (never, past, current). Depending on the model, additional adjustments were included for: BMI (<20.0, 20.0 to 24.9, 25.0 to 29.9, 30.0 to 34.9, 35+ kg/m^2^); strenuous physical activity (rarely/never, at most once per week, or more than once per week); and any activity (rarely/never, at most once per week, two to three times per week, or more than three times per week). All adjustment variables were reported at recruitment. Missing data for the adjustment variables (generally <2% for each variable) were assigned to an additional category. When more than two categories were used for risk comparisons, group‐specific (gs) CIs were calculated,^21^ allowing valid comparisons to be made between any two groups. When only two categories were compared or when log‐linear trends in risk were quoted, conventional CIs were used. To account for measurement error in BMI, risks by BMI categories based on self‐reported data were plotted against mean measured BMI values within the same categories.

For each fracture site, age‐specific incidence rates per 100,000 women using 5‐year age groups from 50 to 54 through 75 to 79 years were calculated, as were cumulative absolute risks for BMI, strenuous activity, and any activity, for ages 50 to 84 years for all fracture sites. Category‐specific RRs for each fracture type and exposure were converted to incidence rates by multiplying them by the appropriate age‐specific incidence rate, dividing by a weighted average of all RRs.^22^ A sensitivity analysis was conducted for risk of fracture according to BMI, strenuous activity, and any exercise separated by never/past users and current users of hormone therapy. Because there were dramatic increases with age for some of the fracture sites, we allowed for potential nonproportional hazards through sensitivity analyses considering fracture risk relationships associated with BMI, strenuous activity, and any activity, according to three age bands (50 to 64.9, 65 to 69.9, 70+ years).

## Results

In total, 1,154,821 postmenopausal women were included in our analyses, aged 56.0 (SD 4.8) years on average at baseline (Table 1). Mean alcohol consumption and the proportion of smokers decreased with increasing BMI, whereas the proportion within the lowest fifth of socioeconomic status―and the proportions reporting no physical activity, and never having used HRT―increased with increasing BMI. With increasing frequency of physical activity, mean BMI decreased and mean alcohol consumption increased.

**Table 1:** Characteristics of Postmenopausal Women in the Million Women Study at Recruitment and Follow‐Up According to BMI and Strenuous Physical Activity

	BMI (kg/m^2^)					Strenuous activity			
	<20.0	20.0–24.9	25.0–29.9	30.0–34.9	35+	Rarely/never (inactive)	At most once per week	More than once per week	All women
*Characteristics at baseline*	*n* = 41,785	*n* = 493,662	*n* = 413,866	*n* = 146,079	*n* = 59,429	*n* = 552,793	*n* = 356,399	*n* = 245,629	*n* = 1,154,821
Mean age at recruitment, years (SD)	55.8 (4.9)	55.8 (4.8)	56.3 (4.8)	56.2 (4.8)	55.7 (4.6)	56.4 (4.9)	55.7 (4.7)	55.8 (4.7)	56.0 (4.8)
Mean height, cm (SD)	164.0 (7.1)	162.9 (6.4)	161.6 (6.5)	160.9 (6.7)	159.7 (7.6)	161.6 (6.7)	162.4 (6.5)	162.5 (6.6)	162.0 (6.7)
Mean weight, kg (SD)	50.9 (4.9)	60.8 (5.8)	70.9 (6.7)	82.9 (7.7)	99.0 (12.1)	70.1 (13.5)	68.3 (11.8)	66.8 (11.3)	68.8 (12.6)
Mean BMI, kg/m^2^ (SD)	18.9 (0.9)	22.9 (1.3)	27.1 (1.4)	32.0 (1.4)	38.8 (3.6)	26.9 (5.0)	25.9 (4.3)	25.3 (4.1)	26.2 (4.7)
Mean alcohol, grams/day (SD)	6.3 (8.1)	6.7 (7.8)	5.8 (7.4)	4.6 (6.9)	3.6 (6.3)	5.2 (7.4)	6.2 (7.4)	7.0 (8.1)	5.9 (7.6)
Mean number of children (SD)	1.9 (1.4)	2.1 (1.2)	2.2 (1.3)	2.4 (1.4)	2.4 (1.5)	2.2 (1.3)	2.1 (1.2)	2.1 (1.3)	2.2 (1.3)
Current smoker (%)	31.8	21.1	18.7	16.5	14.8	24.4	15.4	15.6	19.7
Socioeconomic status: lowest fifth (%)	19.1	15.7	19.3	23.9	29.9	23.9	14.1	14.5	18.9
Never users of HRT (%)	50.6	48.5	49.3	51.9	58.0	50.8	48.9	48.7	49.8
No activity (%)	20.1	16.3	21.7	29.5	37.7	–	–	–	21.1
No strenuous activity (%)	47.1	41.8	49.2	57.9	64.7	–	–	–	47.9
									
*Follow‐up for fracture incidence*									
Woman‐years of follow‐up (in millions)	5.74	4.46	1.56	1.56	0.62	5.90	3.84	2.65	12.39
Incident lower leg fractures (*n*)	123	1230	1183	472	191	1622	905	672	3199
Incident femur (not neck) fractures (*n*)	65	414	396	183	108	737	251	178	1166
Incident forearm (not wrist) fractures (*n*)	161	1324	960	349	132	1460	833	633	2926
Incident humerus fractures (*n*)	207	1939	1765	699	321	2701	1345	885	4931
Incident ankle fractures (*n*)	192	3484	4122	1685	609	4896	3056	2140	10,092
Incident wrist fractures (*n*)	764	7747	5345	1513	514	7574	4787	3522	15,883
Incident neck of femur fractures (*n*)	854	4839	2978	887	329	5867	2398	1622	9887

Women with missing values were excluded when calculating the means or percentages for that given variable.

**Figure 1:**
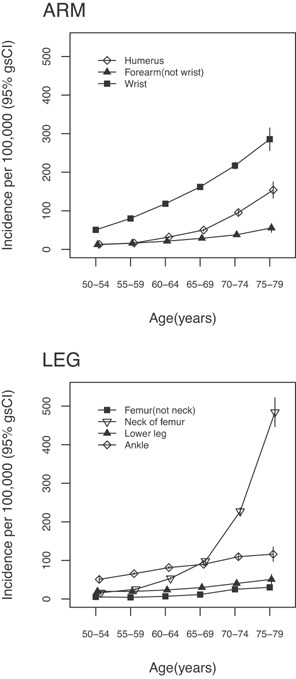
Age‐specific incidence per 100,000 per year (95% gsCI) of arm (wrist, forearm, and humerus) and leg (femur, lower leg, ankle, and hip) fractures among postmenopausal women.

After a mean follow‐up of approximately 11 years per woman, 44,388 women had at least one fracture of the humerus, forearm, wrist, neck of the femur, femur (not neck), lower leg, or ankle. Of these women, 7.6% had more than one fracture during their first hospital admission for any of these fractures. There were 4931 women with a first humerus fracture; 2926 with a first forearm fracture; 15,883 with a first wrist fracture; 9887 with a first fracture of the neck of the femur; 1166 with a first fracture of the femur (not neck); 3199 with a first lower leg fracture; and 10,092 with a first ankle fracture. Age‐specific incidence rates measured in 5‐year age bands increased gradually with age for fractures of forearm, femur (not neck), lower leg, and ankle; and there was a steeper increase in the risk of humerus, neck of the femur, and wrist fracture with increasing age (Fig. 1, Supporting tables 1 and 2). The cumulative absolute risks over 35 years, from ages 50 to 84 years, were 3.0 (95% CI, 2.7 to 3.4)% for humerus fracture; 1.2 (95% CI, 1.0 to 1.4)% for forearm fracture; 6.3 (95% CI, 5.9 to 6.7)% for wrist fracture; 9.1 (95% CI, 8.4 to 9.8)% for neck of the femur fracture; 0.7 (95% CI, 0.6 to 0.9)% for femur (not neck) fracture; 1.3 (95% CI, 1.1 to 1.5)% for lower leg fracture; and 3.2 (95% CI, 2.9 to 3.4)% for ankle fracture.

When compared with women with desirable BMI (20.0 to 24.9 kg/m^2^), having a higher BMI was associated with a lower risk of neck of the femur, forearm, and wrist fractures, but a higher risk of fractures of the humerus, lower leg, ankle, and femur (not neck). Compared with women with desirable BMI, having a lower BMI was associated with a higher risk of fracture at all sites except the ankle (*p* < 0.001; Tables 2 and 3).

**Table 2:** Adjusted Relative Risks of Femur (Not Neck), Neck of Femur, Lower Leg, and Ankle Fracture in Postmenopausal Women by BMI and Physical Activity

		Femur (not neck)			Neck of femur			Lower leg			Ankle		
	Population at risk *n* = 1,154,821	Incident cases *n* = 1166	Relative risk minimally adjusted^b^	Relative risk fully adjusted^b^ RR (95% gsCI)	Incident cases *n* = 9887	Relative risk minimally adjusted^a^	Relative risk fully adjusted^b^ RR (95% gsCI)	Incident cases *n* = 3199	Relative risk minimally adjusted^a^	Relative risk fully adjusted^b^ RR (95% gsCI)	Incident cases *n* = 10,092	Relative risk minimally adjusted^a^	Relative risk fully adjusted^b^ RR (95% gsCI)
*BMI (kg/m^2^) (mean measured)*													
<20.0 (20.40)	41,785	65	1.91	1.73 (1.35–2.21)	854	2.13	1.87 (1.74–2.00)	123	1.21	1.14 (0.95–1.36)	192	0.67	0.65 (0.57–0.75)
20.0–24.9 (24.20)	493,662	414	1.00	1.00 (0.90–1.11)	4839	1.00	1.00 (0.97–1.03)	1230	1.00	1.00 (0.94–1.06)	3484	1.00	1.00 (0.97–1.04)
25.0–29.9 (28.58)	413,866	396	1.08	1.04 (0.94–1.15)	2978	0.68	0.68 (0.66–0.71)	1183	1.11	1.12 (1.06–1.19)	4122	1.38	1.42 (1.38–1.46)
30.0–34.9 (33.21)	146,079	183	1.42	1.25 (1.08–1.45)	887	0.58	0.55 (0.51–0.58)	472	1.26	1.24 (1.13–1.36)	1685	1.60	1.67 (1.59–1.75)
35+ (38.52)	59,429	108	2.22	1.74 (1.43–2.12)	329	0.58	0.50 (0.45–0.56)	191	1.29	1.21 (1.04–1.40)	609	1.47	1.53 (1.41–1.66)
*p* (heterogeneity)				<0.001			<0.001			<0.001			<0.001
													
*Strenuous activity*													
Rarely/never (inactive)	552,793	737	1.00	1.00 (0.91–1.10)	5867	1.00	1.00 (0.97–1.03)	1622	1.00	1.00 (0.94–1.07)	4896	1.00	1.00 (0.96–1.04)
At most once per week	356,399	251	0.58	0.73 (0.64–0.82)	2398	0.72	0.79 (0.76–0.82)	905	0.90	0.99 (0.93–1.06)	3056	1.00	1.03 (1.00–1.07)
> once per week	245,629	178	0.58	0.68 (0.58–0.80)	1622	0.69	0.71 (0.67–0.74)	672	0.96	1.03 (0.95–1.12)	2140	1.00	1.03 (0.99–1.08)
*p* (heterogeneity)				<0.001			<0.001			0.7			0.4
													
*Any activity*	*n* = 1,045,222	*n* = 1018			*n* = 8625			*n* = 2839			*n* = 9034		
Rarely/never (inactive)	220,982	332	1.00	1.00 (0.88–1.14)	2334	1.00	1.00 (0.95–1.05)	671	1.00	1.00 (0.91–1.10)	1964	1.00	1.00 (0.95–1.05)
At most once per week	250,970	189	0.54	0.69 (0.60–0.80)	1741	0.72	0.84 (0.80–0.88)	636	0.87	0.91 (0.84–0.99)	2061	0.95	0.95 (0.91–0.99)
Two to three times per week	248,189	177	0.48	0.67 (0.58–0.78)	1715	0.65	0.80 (0.76–0.84)	570	0.76	0.81 (0.74–0.88)	2070	0.94	0.95 (0.91–1.00)
> three times per week	325,081	320	0.64	0.87 (0.77–0.98)	2835	0.79	0.88 (0.85–0.91)	962	0.96	1.00 (0.94–1.07)	2939	1.00	1.04 (1.00–1.09)
*p* (heterogeneity)				<0.001			<0.001			<0.001			0.05

BMI = body mass index; gsCI = group‐specific confidence interval; RR = relative risk.

^a^ Adjusted for study region, age, and socioeconomic status.

^b^ Adjusted for study region, age, socioeconomic status, smoking, alcohol consumption, parity, use of hormone therapy, height, heart disease/thrombosis, diabetes mellitus, thyroid disease, rheumatoid arthritis/osteoarthritis, and BMI (for adjustment of strenuous activity and any activity), or strenuous activity (for adjustment of BMI and any activity), or any activity (for adjustment of BMI and strenuous activity).

**Table 3:** Adjusted Relative Risks of Forearm (Not Wrist), Wrist, and Humerus Facture in Postmenopausal Women by BMI and Physical Activity

		Forearm (not wrist)			Wrist			Humerus		
	Population at risk *n* = 1,154,821	Incident cases *n* = 2926	Relative risk minimally adjusted^a^	Relative risk fully adjusted^b^ RR (95% gsCI)	Incident cases *n* = 15,883	Relative risk minimally adjusted^a^	Relative risk fully adjusted^b^ RR (95% gsCI)	Incident cases *n* = 4931	Relative risk minimally adjusted^a^	Relative risk fully adjusted^b^ RR (95% gsCI)
*BMI* (*kg/m^2^*) (*mean measured*)										
<20.0 (20.40)	41,785	161	1.48	1.42 (1.22–1.67)	764	1.21	1.19 (1.11–1.28)	207	1.30	1.20 (1.04–1.37)
20.0–24.9 (24.20)	493,662	1324	1.00	1.00 (0.94–1.06)	7747	1.00	1.00 (0.98–1.02)	1939	1.00	1.00 (0.95–1.05)
25.0–29.9 (28.58)	413,866	960	0.84	0.85 (0.80–0.90)	5345	0.79	0.80 (0.77–0.82)	1765	1.02	1.04 (0.99–1.09)
30.0–34.9 (33.21)	146,079	349	0.87	0.87 (0.78–0.96)	1513	0.64	0.64 (0.61–0.67)	699	1.15	1.14 (1.06–1.23)
35+ (38.52)	59,429	132	0.84	0.82 (0.69–0.98)	514	0.56	0.56 (0.51–0.61)	321	1.39	1.31 (1.17–1.47)
*p* (heterogeneity)				<0.001			<0.001			<0.001
										
*Strenuous activity*										
Rarely/never (inactive)	552,793	1460	1.00	1.00 (0.94–1.07)	7574	1.00	1.00 (0.97–1.03)	2701	1.00	1.00 (0.95–1.05)
At most once per week	356,399	833	0.92	0.92 (0.85–0.98)	4787	1.03	1.00 (0.98–1.03)	1345	0.85	0.91 (0.86–0.96)
More than once per week	245,629	633	1.00	0.98 (0.90–1.06)	3522	1.09	1.03 (0.99–1.06)	885	0.80	0.83 (0.77–0.89)
*p* (heterogeneity)				0.2			0.5			<0.001
										
*Any activity*	*n* = 1,045,222	*n* = 2577			*n* = 14,262			*n* = 4340		
Rarely/never (inactive)	220,982	555	1.00	1.00 (0.91–1.10)	2938	1.00	1.00 (0.96–1.04)	1046	1.00	1.00 (0.93–1.07)
At most once per week	250,970	587	0.96	0.98 (0.90–1.07)	3120	0.97	0.93 (0.90–0.97)	948	0.86	0.97 (0.90–1.03)
Two to three times per week	248,189	574	0.91	0.90 (0.83–0.98)	3422	1.02	0.94 (0.91–0.97)	925	0.79	0.93 (0.87–1.00)
More than three times per week	325,081	861	1.02	0.97 (0.91–1.05)	4782	1.08	0.96 (0.93–0.99)	1421	0.90	1.03 (0.97–1.09)
*p* (heterogeneity)				0.4			0.1			0.1

BMI = body mass index; gsCI = group‐specific confidence interval; RR = relative risk.

^a^ Adjusted for study region, age, and socioeconomic status.

^b^ Adjusted for study region, age, socioeconomic status, smoking, alcohol consumption, parity, use of hormone therapy, height, heart disease/thrombosis, diabetes mellitus, thyroid disease, rheumatoid arthritis/osteoarthritis, and BMI (for adjustment of strenuous activity and any activity), or strenuous activity (for adjustment of BMI and any activity), or any activity (for adjustment of BMI and strenuous activity).

**Figure 2:**
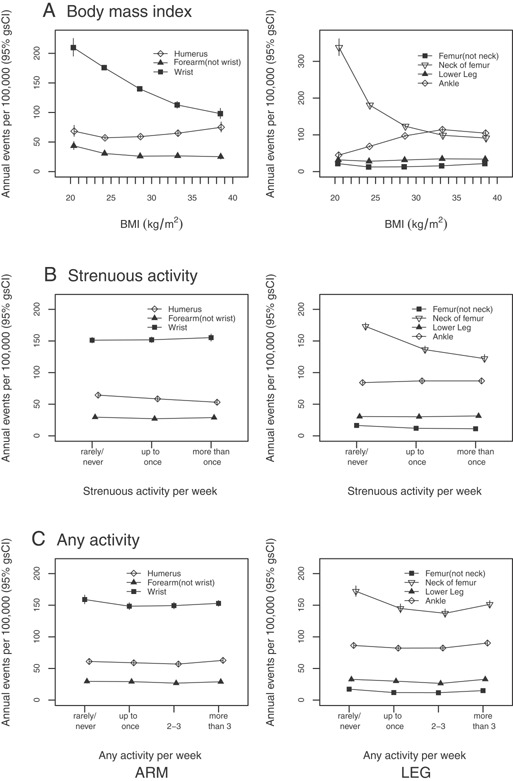
Adjusted absolute risks per 100,000 per year of arm (wrist, forearm, and humerus) and leg (femur, lower leg, ankle, and hip) fractures in postmenopausal women, by BMI, strenuous physical activity, and any activity. For BMI plots, risk estimates are plotted against the mean measured BMI value within each category but offlaid slightly.

RRs of fractures of the femur (both neck and non‐neck) and humerus were lower in women reporting regular strenuous physical activity (*p* < 0.001; Tables 2 and 3; Fig. 2). No significant associations were seen between strenuous activity and risk of lower leg, ankle, forearm, or wrist fracture (*p* ≥ 0.2). When associations with any activity were considered, there was evidence of U‐shaped associations (with the lowest risks observed in women who reported activity two to three times per week) for neck of the femur, femur (not neck), and lower leg fracture (*p* < 0.001), but not for ankle, forearm, wrist, and humerus fracture (*p* ≥ 0.05; Tables 2 and 3; Fig. 2). There was little evidence for interactions between BMI and physical activity for fracture risk (Supplementary tables 3 and 4).

Sensitivity analyses that separately examined current users and never/past users of menopausal HRT (Supporting Tables  5 and 6), as well as subgroups by age at hospital admission (Supporting Tables  7–12), showed generally similar relationships to those in the main analysis for fracture risk by BMI, strenuous activity, and any activity.

## Discussion

In this prospective, population‐based study of over 1 million postmenopausal women, older age was associated with increased rates of fracture at all sites, with especially steep increases with age for fractures of the humerus, wrist, and neck of the femur. In comparison with women with desirable BMI (20.0 to 24.9 kg/m^2^), higher BMI was associated with a decreased risk of wrist, forearm, and neck of the femur fracture, and an increased risk of humerus, lower leg, ankle, and non‐neck femur fractures. Strenuous activity was significantly associated with a reduced risk of humerus and femur (both neck and non‐neck) fractures.

This is the largest prospective study to examine relationships between BMI and fracture risk at multiple sites in postmenopausal women and our results indicate that the relationship with BMI differed according to fracture site. While there is considerable evidence that high BMI is associated with a reduced risk of neck of femur fracture,^3^, ^5^, ^23^ small sample sizes and/or short follow‐up time have limited the ability to draw clear conclusions about the relationships with BMI at other fracture sites. One prospective study of 52,939 postmenopausal women, with a 3‐year follow‐up period, showed that BMI was inversely associated with risk of wrist and neck of the femur fractures, was positively associated with risk of ankle fractures, but showed no significant associations with upper arm/shoulder fractures.^24^ Another prospective study of Spanish women over the age of 50 years found that in comparison with normal/underweight women, obese women had an increased risk of proximal humerus fractures and a decreased risk of neck of femur fractures; however, there was no significant association between BMI and lower leg or wrist fractures.^25^

This is also the largest prospective study examining the associations between physical activity and the risk of site‐specific fractures in postmenopausal women. We found that strenuous activity was associated with a decreased risk of humerus and femur fracture (both neck and non‐neck), but not at other sites. For any activity, there was a suggestion of U‐shaped associations for risk of femur fracture (both neck and non‐neck) and lower leg, but not at other sites. Comparisons with previous prospective studies are hampered by a lack of standardized physical activity measures across studies and small sample sizes. Increased physical activity has consistently been associated with a decreased risk of neck of femur fracture.^3^, ^5^, ^26^ A higher risk of wrist fracture with physical activity has been found in some studies^27^, ^28^ but not in all.^29^ One study showed an increased risk of ankle fracture with activity,^30^ whereas two others reported no significant associations.^27^, ^28^ There have also been reports of no significant association between physical activity and fractures of the humerus^27^, ^31^ and forearm.^31^ For all of these studies, numbers were small and null findings may have resulted from a lack of power.

How fat distribution and obesity impact bone health and fracture risk at different sites is not fully understood. Increased adiposity is assumed to cushion the impact on some bones while falling; however, the effects of fat on bone mineral density may depend not just on adiposity overall but on factors related to fat distribution.^32^ Peripheral fat (subcutaneous fat found directly under the skin) is a major source for endogenous estrogen that increases bone mineral density in postmenopausal women,^33^, ^34^ whereas ectopic fat (visceral fat that accumulates around the abdominal area and many organs of the body) is associated with insulin resistance that may have negative effects on bone health.^32^, ^35^, ^36^ In our study, women with the lowest BMI (<20) had a higher risk for fractures at all sites except the ankle, when compared with women with desirable BMI (20.0 to 24.9 kg/m^2^). Both low BMI and an increase in fracture risk might be a consequence of frailty or another disease^37^ and hence these findings may reflect reverse causation.

The relationship between physical activity and fracture is complex and the evidence on potential mechanisms is sparse, especially when individual fracture sites are considered. Physical activity may attenuate age‐related bone loss through improving bone mineral density and/or reducing bone loss.^38^, ^39^, ^40^ Regular physical activity may protect against falls through improved balance, muscle strength, and coordination.^2^, ^41^ However, while participating in regular physical activity, people are at an increased risk of falls that may lead to injury.^2^, ^42^ The “fear of falling” may lead to the adoption of more cautious physical activity behaviors that could in turn increase fall risk.^39^ The lack of associations with physical activity for some fracture sites could in principle mask these competing influences on fracture risk, or their effects on fracture risk at these sites may be modest.

The primary strengths of this study are its large population sample and prospective design with an average follow‐up of 11 years. Whereas having access to objective hospital records of fracture is a strength, a potential limitation is that fractures not leading to day‐case or overnight admission were not captured. Almost all neck of the femur and femur (not neck) fractures result in an overnight hospital stay,^1^, ^37^ and many reduction procedures and/or anesthetics given in relation to fracture result in a day‐case or overnight stay,^43^ but some relatively minor fractures may not be included in hospital data.^44^ We used BMI as a marker of adiposity because body fatness generally accounts for most of the population variation in BMI, although it has been reported that BMI is a suboptimal marker of body fat composition as measured by DXA in older adults.^45^ Age‐related changes in lean mass and physical function,^46^ and the patterns of change in BMI over time differ among individuals.^47^ Overall in this cohort, validation studies indicate that BMI is relatively stable over time, and individual changes during follow‐up are not likely to be a source of substantial bias.^15^ Physical activity also has good repeatability, although this declines slightly over time.^17^

Another limitation of this study was not having a measure of bone mineral density.^23^ Fracture risk is higher in frail individuals with multiple morbidities^42^ because these individuals are less active and may have a low BMI as a result of their illness. On the other hand, having a high BMI is significantly associated with a number of comorbidities, including type 2 diabetes and heart disease.^32^ We excluded women with self‐reported osteoporosis and other known prior morbidities to eliminate the possibility that these conditions altered behavior; however, we cannot rule out the possibility that some conditions may not have been reported by all participants. Our analyses were censored at the first occurrence of any fracture, to account for the increased risk of subsequent fracture reported among women with a prior fracture.^14^ Because of these exclusions, the absolute rates quoted here may not be directly comparable with those published previously.

One of the most commonly reported health conditions in postmenopausal women is osteoporosis.^48^, ^49^ Although an estimated 30% of women have osteoporosis,^50^ it has been reported that approximately 50% of women over the age of 50 years will have a fracture in their lifetimes,^1^, ^51^, ^52^ and these conditions increase markedly with age as our results show. Drugs that increase bone mineral density are available to reduce the risk of fracture; however, there is lack of evidence about their long‐term effects^53^ and therefore it is important that behavioral strategies are investigated.

In conclusion, risk factors for fracture in postmenopausal women differ substantially by fracture site. In comparison with women of desirable BMI (20.0 to 24.9 kg/m^2^), increased BMI was associated with a reduced risk of neck of the femur, forearm, and wrist fractures, with the opposite found for humerus, femur (not neck), lower leg, and ankle fractures. Strenuous activity was associated with a reduced risk of femur (both neck and non‐neck) and humerus fractures.

## Disclosures

VB is a nonexecutive director of the Medicines and Healthcare Products Regulatory Agency. JL was supported by a Sir Frederick Banting and Charles Best Canada Graduate Scholarship‐Master's from CIHR and the Psychosocial Oncology Research Training Program (PORT)‐Master's, as well as by the Michael Smith Foreign Study Supplements Program. BJC acknowledges support from the BHF Centre of Research Excellence, Oxford, England. JG, GKR, and MEGA report no conflicts of interest. The study sponsors did not influence the conduct of the study or the preparation of this article.

## Supporting information

Supporting Information.
