# Supplementary material for: The Effects of Age, Adiposity, and Physical Activity on the Risk of Seven Site‐Specific Fractures in Postmenopausal Women
Source: J Bone Miner Res. 2016 May 5;31(8):1559–68. doi: 10.1002/jbmr.2826 (PMC4973709; doi:10.1002/jbmr.2826)
Supplement: Supplementary file 1 — Supporting Information. [file JBMR-31-1559-s001.docx]

**Supporting Acknowledgments**

**The Million Women Study Advisory Committee:** Emily Banks, Valerie Beral, Lucy Carpenter, Carol Dezateux, Jane Green, Julietta Patnick, Richard Peto, Cathie Sudlow.

**NHS Breast Screening Centres collaborating in the Million Women Study** (in alphabetical order): Avon, Aylesbury, Barnsley, Basingstoke, Bedfordshire & Hertfordshire, Cambridge & Huntingdon, Chelmsford & Colchester, Chester, Cornwall, Crewe, Cumbria, Doncaster, Dorset, East Berkshire, East Cheshire, East Devon, East of Scotland, East Suffolk, East Sussex, Gateshead, Gloucestershire, Great Yarmouth, Hereford & Worcester, Kent (Canterbury, Rochester, Maidstone), Kings Lynn, Leicestershire, Liverpool, Manchester, Milton Keynes, Newcastle, North Birmingham, North East Scotland, North Lancashire, North Middlesex, North Nottingham, North of Scotland, North Tees, North Yorkshire, Nottingham, Oxford, Portsmouth, Rotherham, Sheffield, Shropshire, Somerset, South Birmingham, South East Scotland, South East Staffordshire, South Derbyshire, South Essex, South Lancashire, South West Scotland, Surrey, Warrington Halton St Helens & Knowsley, Warwickshire Solihull & Coventry, West Berkshire, West Devon, West London, West Suffolk, West Sussex, Wiltshire, Winchester, Wirral and Wycombe.

**The Million Women Study Coordinating Centre staff:** Hayley Abbiss, Simon Abbott, Rupert Alison, Naomi Allen, Miranda Armstrong, Krys Baker, Angela Balkwill, Emily Banks, Isobel Barnes, Valerie Beral, Judith Black, Roger Blanks, Kathryn Bradbury, Anna Brown, Benjamin Cairns, Karen Canfell, Dexter Canoy, Andrew Chadwick, Barbara Crossley, Francesca Crowe, Dave Ewart, Sarah Ewart, Lee Fletcher, Sarah Floud, Toral Gathani, Laura Gerrard, Adrian Goodill, Jane Green, Lynden Guiver, Michal Hozak, Isobel Lingard, Sau Wan Kan, Oksana Kirichek, Nicky Langston, Bette Liu, Kath Moser, Kirstin Pirie, Gillian Reeves, Keith Shaw, Emma Sherman, Helena Strange, Sian Sweetland, Sarah Tipper, Ruth Travis, Lyndsey Trickett, Lucy Wright, Owen Yang, Heather Young.

| **Supporting Table 1.** Age specific incidence rates per 100,000 per year for leg fractures: femur (not neck), neck of femur, lower leg, and ankle in post-menopausal women | | | | | | | | | |
| --- | --- | --- | --- | --- | --- | --- | --- | --- | --- |
|  |  | **Femur (not neck)** | | **Neck of femur** | | **Lower leg** | | **Ankle** | |
|  | **Person Years** | **Incident**  **cases** | **Incidence rate per 100,000**  **(95% CI)** | **Incident**  **cases** | **Incidence rate per 100,000**  **(95% CI)** | **Incident**  **cases** | **Incidence rate per 100,000**  **(95% CI)** | **Incident**  **cases** | **Incidence rate per 100,000**  **(95% CI)** |
| **Age (years)** |  |  |  |  |  |  |  |  |  |
| 50-54 | 469,406 | 20 | 5.4 (2.4-8.5) | 86 | 16.2 (11.9-20.6) | 79 | 19.8 (14.1-25.6) | 250 | 50.8 (42.9-58.7) |
| 55-59 | 3,376,760 | 148 | 4.3 (3.6-5.0) | 873 | 25.3 (23.6-26.9) | 669 | 19.6 (18.1-21.1) | 2250 | 65.6 (62.9-68.4) |
| 60-64 | 4,178,337 | 290 | 7.0 (6.2-7.8) | 2222 | 53.4 (51.1-55.6) | 981 | 23.5 (22.0-24.9) | 3401 | 81.5 (78.7-84.2) |
| 65-69 | 2,824,762 | 313 | 11.5 (10.2-12.8) | 2662 | 98.2 (94.4-102.0) | 838 | 29.7 (27.7-31.8) | 2540 | 89.8 (86.3-93.4) |
| 70-74 | 1,273,714 | 292 | 25.2 (22.1-28.2) | 2665 | 227.4 (218.3-236.5) | 501 | 40.3 (36.6-44.0) | 1357 | 109.6 (103.5-115.8) |
| 75-79 | 231,944 | 80 | 30.2 (22.6-37.8) | 1027 | 484.2 (446.9-521.4) | 112 | 50.8 (38.2-63.4) | 254 | 1116.0 (97.6-134.9) |

| **Supporting Table 2.** Age specific incidence rates per 100,000 per year for arm fractures: forearm (not wrist), wrist, and humerus in post-menopausal women | | | | | | | |
| --- | --- | --- | --- | --- | --- | --- | --- |
|  |  | **Forearm (not wrist)** | | **Wrist** | | **Humerus** | |
|  | **Person Years** | **Incident**  **cases** | **Incidence rate per 100,000 (95% CI)** | **Incident**  **cases** | **Incidence rate per 100,000 (95% CI)** | **Incident**  **cases** | **Incidence rate per 100,000 (95% CI)** |
| **Age (years)** |  |  |  |  |  |  |  |
| 50-54 | 469,406 | 62 | 12.3 (8.2-16.3) | 272 | 50.7 (43.4-57.9) | 60 | 12.9 (8.7-17.1) |
| 55-59 | 3,376,760 | 544 | 15.9 (14.6-17.3) | 2761 | 80.2 (77.2-83.2) | 572 | 16.4 (15.1-17.8) |
| 60-64 | 4,178,337 | 893 | 21.4 (20.0-22.8) | 4938 | 118.3 (115.0-121.6) | 1334 | 32.0 (30.3-33.7) |
| 65-69 | 2,824,762 | 794 | 28.8 (26.7-30.8) | 4487 | 161.9 (157.1-166.7) | 1387 | 50.1 (47.5-52.8) |
| 70-74 | 1,273,714 | 471 | 37.9 (34.3-41.5) | 2674 | 217.2 (208.5-225.8) | 1158 | 95.6 (89.7-101.4) |
| 75-79 | 231,944 | 134 | 55.6 (44.2-67.0) | 627 | 285.6 (256.4-314.8) | 337 | 153.8 (132.9-174.7) |

| **Supporting Table 3.** Adjusted^a^ relative risks of femur (not neck), neck of femur, lower leg, and ankle fractures in women, cross-classified by both BMI and strenuous physical activity, and BMI and any activity | | | | | | | | | |
| --- | --- | --- | --- | --- | --- | --- | --- | --- | --- |
|  | **Strenuous Physical Activity** | | | | **Any activity** | | | | |
| **BMI (kg/m^2^)** | **Rarely/never active** | **At most once per week**  **RR (gsCI)** | **More than once per week**  **RR (gsCI)** | ***P* _for interaction_** | **Rarely/never active** | **At most once per week**  **RR (gsCI)** | **2-3 times per week**  **RR (gsCI)** | **More than 3 times per week**  **RR (gsCI)** | ***P* _for interaction_** |
| **Femur (not neck)** |  |  |  |  |  |  |  |  |  |
| <25.0 | 1.00 (0.88-1.13)^c^ | 0.67 (0.56-0.81) | 0.61 (0.49-0.76) | .13^b^ | 1.00 (0.82-1.22)^c^ | 0.58 (0.45-0.75) | 0.56 (0.44-0.71) | 0.81 (0.69-0.96) | .52^c^ |
| 25.0-29.9 | 0.94 (0.83-1.08) | 0.61 (0.49-0.76) | 0.72 (0.56-0.93) |  | 0.85 (0.69-1.03) | 0.59 (0.46-0.75) | 0.67 (0.53-0.85) | 0.81 (0.67-0.98) |  |
| 30.0+ | 1.18 (1.01-1.38) | 1.16 (0.90-1.50) | 0.80 (0.54-1.18) |  | 1.16 (0.95-1.42) | 0.93 (0.72-1.21) | 0.79 (0.56-1.10) | 0.91 (0.68-1.21) |  |
|  |  |  |  |  |  |  |  |  |  |
| **Neck of femur** |  |  |  |  |  |  |  |  |  |
| <25.0 | 1.00 (0.96-1.04)^c^ | 0.75 (0.71-0.79) | 0.67 (0.63-0.72) | .04^b^ | 1.00 (0.94-1.06)^c^ | 0.81 (0.76-0.87) | 0.73 (0.68-0.78) | 0.84 (0.80-0.88) | .05^c^ |
| 25.0-29.9 | 0.61 (0.58-0.64) | 0.49 (0.46-0.53) | 0.46 (0.42-0.50) |  | 0.58 (0.53-0.62) | 0.49 (0.45-0.53) | 0.51 (0.47-0.55) | 0.54 (0.50-0.58) |  |
| 30.0+ | 0.47 (0.43-0.50) | 0.43 (0.38-0.48) | 0.36 (0.30-0.43) |  | 0.46 (0.41-0.50) | 0.39 (0.34-0.44) | 0.39 (0.34-0.46) | 0.47 (0.42-0.53) |  |
|  |  |  |  |  |  |  |  |  |  |
| **Lower Leg** |  |  |  |  |  |  |  |  |  |
| <25.0 | 1.00 (0.92-1.09)^c^ | 0.95 (0.86-1.05) | 0.99 (0.89-1.11) | .52^b^ | 1.00 (0.87-1.15)^c^ | 0.88 (0.77-1.01) | 0.80 (0.71-0.91) | 1.01 (0.92-1.11) | .52^c^ |
| 25.0-29.9 | 1.07 (0.98-1.16) | 1.12 (1.01-1.24) | 1.07 (0.93-1.22) |  | 1.09 (0.96-1.24) | 1.03 (0.91-1.17) | 0.89 (0.78-1.02) | 1.09 (0.98-1.22) |  |
| 30.0+ | 1.17 (1.05-1.30) | 1.11 (0.94-1.31) | 1.40 (1.16-1.70) |  | 1.20 (1.04-1.38) | 1.10 (0.93-1.29) | 0.95 (0.78-1.16) | 1.23 (1.04-1.46) |  |
|  |  |  |  |  |  |  |  |  |  |
| **Ankle** |  |  |  |  |  |  |  |  |  |
| <25.0 | 1.00 (0.95-1.05)^c^ | 0.97 (0.91-1.03) | 1.03 (0.96-1.10) | .05^b^ | 1.00 (0.92-1.09)^c^ | 0.88 (0.81-0.95) | 0.91 (0.85-0.98) | 0.96 (0.92-1.03) | .58^c^ |
| 25.0-29.9 | 1.40 (1.34-1.47) | 1.48 (1.40-1.56) | 1.49 (1.39-1.60) |  | 1.35 (1.25-1.45) | 1.31 (1.22-1.40) | 1.30 (1.21-1.39) | 1.47 (1.39-1.56) |  |
| 30.0+ | 1.63 (1.53-1.72) | 1.80 (1.67-1.95) | 1.53 (1.36-1.71) |  | 1.52 (1.40-1.65) | 1.54 (1.41-1.67) | 1.49 (1.35-1.65) | 1.61 (1.47-1.76) |  |

BMI indicates body mass index; gsCI, group-specific confidence interval for RR; and RR, relative risk.

^a^ Adjusted for study region, age, socio-economic status, smoking, alcohol consumption, use of hormone therapy, height, heart disease/thrombosis, diabetes mellitus, thyroid disease, rheumatoid arthritis/osteoarthritis, and exercise

^b^ Likelihood ratio test for interaction between categories of BMI and strenuous activity
^c^ Likelihood ratio test for interaction between categories of BMI and any activity.

| **Supporting Table 4.** Adjusted^a^ relative risks of forearm (not wrist), wrist, and humerus fractures in women, cross-classified by both BMI and strenuous physical activity, and BMI and any activity | | | | | | | | | |
| --- | --- | --- | --- | --- | --- | --- | --- | --- | --- |
|  | **Strenuous Physical Activity** | | | | **Any Activity** | | | | |
| **BMI (kg/m^2^)** | **Rarely/never active** | **At most once per week**  **RR (gsCI)** | **More than once per week**  **RR (gsCI)** | ***P* _for interaction_** | **Rarely/never active** | **At most once per week**  **RR (gsCI)** | **2-3 times per week**  **RR (gsCI)** | **More than 3 times per week RR (gsCI)** | ***P* _for interaction_** |
| **Forearm (not wrist)** |  |  |  |  |  |  |  |  |  |
| <25.0 | 1.00 (0.92-1.08) | 0.93 (0.84-1.02) | 1.02 (0.92-1.13) | .54^b^ | 1.00 (0.87-1.15) | 1.09 (0.96-1.23) | 0.86 (0.76-0.97) | 1.10 (1.00-1.20) | .03^c^ |
| 25.0-29.9 | 0.83 (0.75-0.91) | 0.78 (0.69-0.88) | 0.80 (0.63-0.93) |  | 0.88 (0.76-1.02) | 0.85 (0.74-0.98) | 0.90 (0.79-1.03) | 0.82 (0.72-0.93) |  |
| 30.0+ | 0.89 (0.79-1.00) | 0.72 (0.59-0.87) | 0.72 (0.55-0.93) |  | 0.92 (0.78-1.09) | 0.77 (0.63-0.93) | 0.85 (0.68-1.05) | 0.75 (0.60-0.93) |  |
|  |  |  |  |  |  |  |  |  |  |
| **Wrist** |  |  |  |  |  |  |  |  |  |
| <25.0 | 1.00 (0.97-1.03) | 0.99 (0.95-1.03) | 1.03 (0.99-1.08) | .83^b^ | 1.00 (0.94-1.06) | 0.90 (0.86-0.95) | 0.92 (0.88-0.96) | 0.95 (0.92-0.99) | .88^c^ |
| 25.0-29.9 | 0.76 (0.75-0.81) | 0.79 (0.75-0.83) | 0.80 (0.75-0.85) |  | 0.76 (0.71-0.81) | 0.73 (0.69-0.77) | 0.73 (0.69-0.78) | 0.74 (0.70-0.78) |  |
| 30.0+ | 0.61 (0.58-0.65) | 0.61 (0.56-0.67) | 0.59 (0.53-0.67) |  | 0.59 (0.54-0.64) | 0.56 (0.51-0.62) | 0.55 (0.49-0.61) | 0.60 (0.54-0.66) |  |
|  |  |  |  |  |  |  |  |  |  |
| **Humerus** |  |  |  |  |  |  |  |  |  |
| <25.0 | 1.00 (0.94-1.07) | 0.92 (0.85-1.00) | 0.85 (0.77-0.93) | .84^b^ | 1.00 (0.90-1.11) | 0.95 (0.85-1.05) | 0.92 (0.83-1.01) | 0.96 (0.89-1.04) | .76^c^ |
| 25.0-29.9 | 1.03 (0.97-1.10) | 0.90 (0.82-0.99) | 0.84 (0.74-0.94) |  | 0.97 (0.87-1.08) | 0.92 (0.83-1.02) | 0.90 (0.81-1.01) | 1.04 (0.95-1.14) |  |
| 30.0+ | 1.17 (1.08-1.27) | 1.10 (0.96-1.25) | 0.90 (0.75-1.09) |  | 1.08 (0.96-1.22) | 1.09 (0.95-1.24) | 1.01 (0.86-1.18) | 1.23 (1.08-1.41) |  |

BMI indicates body mass index; gsCI, group-specific confidence interval for RR; and RR, relative risk.

^a^ Adjusted for study region, age, socio-economic status, smoking, alcohol consumption, use of hormone therapy, height, heart disease/thrombosis, diabetes mellitus, thyroid disease, rheumatoid arthritis/osteoarthritis, and exercise
^b^ Likelihood ratio test for interaction between categories of BMI and strenuous activity
^c^ Likelihood ratio test for interaction between categories of BMI and any activity.

| **Supporting Table 5.** Sensitivity analyses of hormone therapy for relative risk of femur (not neck), neck of femur, lower leg, and ankle fractures in post-menopausal women according to strenuous physical activity, and any activity ^a^ | | | | | | | | |
| --- | --- | --- | --- | --- | --- | --- | --- | --- |
|  | **Femur (not neck)** | | **Neck of femur** | | **Lower leg** | | **Ankle** | |
|  | **Current users of hormone therapy**  **RR (95%** gsCI**)** | **Never/past hormone therapy use**  **RR (95%** gsCI**)** | **Current users of hormone therapy**  **RR (95%** gsCI**)** | **Never/past hormone therapy use**  **RR (95%** gsCI**)** | **Current users of hormone therapy**  **RR (95%** gsCI**)** | **Never/past hormone therapy use**  **RR (95%** gsCI**)** | **Current users of hormone therapy**  **RR (95%** gsCI**)** | **Never/past hormone therapy use**  **RR (95%** gsCI**)** |
| **BMI (kg/m^2^)** |  |  |  |  |  |  |  |  |
| <20.0 | 1.44 (0.88-2,37) | 1.87 (1.41-2.52) | 1.80 (1.57-2.07) | 1.96 (1.81-2.21) | 0.88 (0.60-1.27) | 1.28 (1.04-1.56) | 0.64 (0.49-0.82) | 0.66 (0.56-0.79) |
| 20.0-24.9 | 1.00 (0.83-1.20) | 1.00 (0.88-1.13) | 1.00 (0.94-1.06) | 1.00 (0.97-1.04) | 1.00 (0.90-1.11) | 1.00 (0.93-1.07) | 1.00 (0.94-1.06) | 1.00 (0.96-1.04) |
| 25.0-29.9 | 1.01 (0.83-1.23) | 1.06 (0.94-1.19) | 0.69 (0.64-0.74) | 0.67 (0.64-0.70) | 1.11 (0.99-1.24) | 1.11 (1.04-1.19) | 1.48 (1.40-1.56) | 1.37 (1.32-1.42) |
| 30.0-34.9 | 1.07 (0.77-1.48) | 1.30 (1.10-1.54) | 0.59 (0.51-0.68) | 0.52 (0.49-0.56) | 1.37 (1.14-1.63) | 1.19 (1.07-1.32_ | 1.95 (1.79-2.13) | 1.54 (1.45-1.63) |
| 35+ | 1.43 (0.88-2.33) | 1.87 (1.51-2.33) | 0.65 (0.51-0.83) | 0.48 (0.42-0.54) | 1.61 (1.21-2.15) | 1.12 (0.94-1.32) | 1.70 (1.44-2.01) | 1.45 (1.32-1.59) |
| *P_heterogeneity_* | .51 | <.001 | <.001 | <.001 | <.05 | <.05 | <.001 | <.001 |
| *P_interaction_* | .75 | | .14 | | <.05 | | <.001 | |
|  |  |  |  |  |  |  |  |  |
| **Strenuous Exercise** |  |  |  |  |  |  |  |  |
| Rarely/never (inactive) | 1.00 (0.82-1.22) | 1.00 (0.83-1.16) | 1.00 (0.93-1.07) | 1.00 (0.96-1.04) | 1.00 (0.88-1.13) | 1.00 (0.93-1.08) | 1.00 (0.94-1.07) | 1.00 (0.96-1.04) |
| At most once per week | 0.83 (0.66-1.04) | 0.66 (0.57-0.77) | 0.81 (0.75-0.87) | 0.77 (0.74-0.81) | 1.09 (0.97-1.23) | 0.95 (0.88-1.03) | 0.94 (0.88-1.01) | 1.06 (1.02-1.11) |
| More than once per week | 0.76 (0.57-1.02) | 0.67 (0.56-0.80) | 0.69 (0.62-0.76) | 0.70 (0.66-0.75) | 1.26 (1.09-1.47) | 0.95 (0.86-1.05) | 1.02 (0.94-1.11) | 1.04 (0.98-1.10) |
| *P_heterogeneity_* | .25 | <.001 | <.001 | <.001 | .06 | .57 | .42 | .12 |
| *P_interaction_* | .51 |  | .56 |  | .34 | .08 | .003 |  |
|  |  |  |  |  |  |  |  |  |
| **Any Activity** |  |  |  |  |  |  |  |  |
| Rarely/never (inactive) | 1.00 (0.76-1.32) | 1.00 (0.86-1.16) | 1.00 (0.90-1.11) | 1.00 (0.95-1.06) | 1.00 (0.83-1.20) | 1.00 (0.90-1.11) | 1.00 (0.91-1.10) | 1.00 (0.94-1.07) |
| At most once per week | 0.70 (0.53-0.91) | 0.67 (0.56-0.80) | 0.85 (0.78-0.94) | 0.84 (0.79-0.89) | 0.92 (0.791.06) | 0.90 (0.82-0.99) | 1.01 (0.93-1.09) | 0.93 (0.88-0.98) |
| 2-3 times per week | 0.48 (0.34-0.67) | 0.70 (0.59-0.83) | 0.93 (0.85-1.02) | 0.75 (0.71-0.809) | 0.71 (0.61-0.84) | 0.84 (0.76-0.93) | 0.96 (0.88-1.04) | 0.95 (0.90-1.00) |
| More than 3 times per week | 0.90 (0.72-1.12) | 0.83 (0.72-0.95) | 1.03 (0.95-1.11) | 0.83 (0.79-0.87) | 0.86 (0.75-0.98) | 1.05 (0.97-1.14) | 1.15 (1.07-1. 23) | 1.00 (0.95-1.04) |
| *P_heterogeneity_* | <.05 | <.05 | <.05 | <.001 | <.05 | <.001 | <.05 | .14 |
| *P_interaction_* | .22 |  | <.05 |  | .62 |  | .19 |  |

BMI indicates body mass index; gsCI, group-specific confidence interval for RR; and RR, relative risk.

^a^ Adjusted for study region, age, socio-economic status, smoking, alcohol consumption, height, heart disease/thrombosis, diabetes mellitus, thyroid disease, rheumatoid arthritis/osteoarthritis, and BMI (for adjustment of strenuous activity and any activity), strenuous activity (for adjustment of BMI and any activity), or any activity (for adjustment of BMI and strenuous activity).

| **Supporting Table 6.** Sensitivity analyses of hormone therapy for relative risk of forearm (not wrist), wrist, and humerus fractures in post-menopausal women according to BMI, strenuous physical activity, and any activity ^a^ | | | | | | | | |
| --- | --- | --- | --- | --- | --- | --- | --- | --- |
|  | **Forearm (not wrist)** | | **Wrist** | | | **Humerus** | | |
|  | **Current users of hormone therapy**  **RR (95%** gsCI**)** | **Never/past hormone therapy use**  **RR (95%** gsCI**)** | **Current users of hormone therapy**  **RR (95%** gsCI**)** | | **Never/past hormone therapy use**  **RR (95%** gsCI**)** | **Current users of hormone therapy**  **RR (95%** gsCI**)** | **Never/past hormone therapy use**  **RR (95%** gsCI**)** | |
| **BMI (kg/m^2^)** |  |  |  | |  |  |  | |
| <20.0 | 1.41 (1.03-1.91) | 1.44 (1.19-1.73) | 1.05 (0.90-1.22) | | 1.00 (1.16-1.37) | 1.25 (0.96-1.63) | 1.19 (1.00-1.40) | |
| 20.0-24.9 | 1.00 (0.90-1.12) | 1.00 (0.94-1.07) | 1.00 (0.95-1.05) | | 1.00 (0.97-1.03) | 1.00 (0.91-1.09) | 1.00 (0.95-1.06) | |
| 25.0-29.9 | 0.99 (0.87-1.11) | 0.79 (0.74-0.85) | 0.80 (0.75-0.84) | | 0.80 (0.77-0.82) | 1.10 (1.00-1.21) | 1.00 (0.95-1.06) | |
| 30.0-34.9 | 0.91 (0.72-1.15) | 0.84 (0.75-0.95) | 0.67 (0.60-0.75) | | 0.63 (0.60-0.67) | 1.25 (1.07-1.47) | 1.11 (1.02-1.21) | |
| 35+ | 0.73 (0.46-1.15) | 0.82 (0.68-0.99) | 0.65 (0.53-0.80) | | 0.54 (0.49-0.60) | 1.52 (1.17-1.98) | 1.25 (1.10-1.43) | |
| *P_heterogeneity_* | .15 | <.001 | <.001 | | <.001 | <.05 | <.001 | |
| *P_interaction_* | .26 |  | .19 |  | | .69 | |  |
|  |  |  |  | |  |  |  | |
| **Strenuous Exercise** |  |  |  | |  |  |  | |
| Rarely/never (inactive) | 1.00 (0.87-1.15) | 1.00 (0.93-1.08) | 1.00 (0.94-1.06) | | 1.00 (0.97-1.03) | 1.00 (0.90-1.11) | 1.00 (0.95-1.06) | |
| At most once per week | 1.15 (1.02-1.30) | 0.85 (0.78-0.92) | 1.03 (0.97-1.09) | | 1.00 (0.96-1.03) | 0.99 (0.90-1.10) | 0.89 (0.84-0.95) | |
| More than once per week | 1.06 (0.90-1.24) | 0.95 (0.86-1.05) | 1.05 (0.98-1.13) | | 1.02 (0.98-1.07) | 0.82 (0.71-0.94) | 0.83 (0.76-0.90) | |
| *P_heterogeneity_* | .33 | <.05 | .60 | | .58 | <.05 | <.001 | |
| *P_interaction_* | <.05 |  | .81 | |  | .52 |  | |
|  |  |  |  | |  |  |  | |
| **Any Activity** |  |  |  | |  |  |  | |
| Rarely/never (inactive) | 1.00 (0.81-1.24) | 1.00 (0.89-1.12) | 1.00 (0.91-1.09) | | 1.00 (0.95-1.05) | 1.00 (0.86-1.16) | 1.00 (0.92-1.09) | |
| At most once per week | 0.92 (0.78-1.09) | 1.01 (0.92-1.11) | 0.85 (0.79-0.92) | | 0.95 (0.91-0.99) | 0.83 (0.73-0.95) | 0.99 (0.92-1.07) | |
| 2-3 times per week | 0.99 (0.84-1.16) | 0.85 (0.77-0.94) | 0.83 (0.77-0.89) | | 0.97 (0.93-1.01) | 0.89 (0.78-1.01) | 0.93 (0.86-1,00) | |
| More than 3 times per week | 1.06 (0.93-1.22) | 0.94 (0.87-1.03) | 0.93 (0.87-0.99) | | 0.97 (0.93-1.00) | 0.95 (0.85-1.07) | 1.06 (0.99-1.13) | |
| *P_heterogeneity_* | .63 | .09 | <.001 | | .53 | .23 | .07 | |
| *P_interaction_* | .11 |  | <.05 | |  | .44 |  | |

BMI indicates body mass index; gsCI, group-specific confidence interval for RR; and RR, relative risk.

^a^ Adjusted for study region, age, socio-economic status, smoking, alcohol consumption, height, heart disease/thrombosis, diabetes mellitus, thyroid disease, rheumatoid arthritis/osteoarthritis, and BMI (for adjustment of strenuous activity and any activity), strenuous activity (for adjustment of BMI and any activity), or any activity (for adjustment of BMI and strenuous activity).

| **Supporting Table 7.** Sensitivity analyses of adjusted^a^ relative risks of femur (not neck), neck of femur, lower leg, and ankle fractures in post-menopausal women according to BMI, stratified by age-bands. | | | | | | | | | | | | |
| --- | --- | --- | --- | --- | --- | --- | --- | --- | --- | --- | --- | --- |
|  | **Femur (not neck)** | | | **Neck of Femur** | | | **Lower Leg** | | | **Ankle** | | |
| **BMI (kg/m^2^)** | **Population at risk** | **Incident cases** | **Adjusted**  **RR (95% gsCI)** | **Population at risk** | **Incident cases** | **Adjusted**  **RR (95% gsCI)** | **Population at risk** | **Incident cases** | **Adjusted**  **RR (95% gsCI)** | **Population at risk** | **Incident cases** | **Adjusted RR (95% gsCI)** |
|  |  |  |  |  |  |  |  |  |  |  |  |  |
| **50-64.9 years** |  |  |  |  |  |  |  |  |  |  |  |  |
| <20.0 | 40 143 | 29 | 1.83 (1.27-2.65) | 40 143 | 336 | 2.11 (1.89-2.35) | 40 142 | 73 | 1.21 (0.96-1.52) | 40 142 | 104 | 0.61 (0.50-0.74) |
| 20.0-24.9 | 476 464 | 168 | 1.00 (0.85-1.17) | 476 468 | 1646 | 1.00 (0.95-1.05) | 476 471 | 684 | 1.00 (0.92-1.08) | 476 466 | 2004 | 1.00 (0.96-1.05) |
| 25.0-29.9 | 398 181 | 145 | 1.01 (0.86-1.18) | 398 184 | 857 | 0.62 (0.58-0.67) | 398 182 | 626 | 1.15 (1.07-1.25) | 398 188 | 2373 | 1.51 (1.45-1.57) |
| 30.0-34.9 | 141 028 | 67 | 1.19 (0.93-1.51) | 141 028 | 231 | 0.43 (0.38-9.50) | 141 028 | 240 | 1.23 (1.08-1.40) | 141 033 | 1024 | 1.87 (1.76-1.99) |
| 35+ | 57 956 | 46 | 1.71 (1.26-2.31) | 57 956 | 101 | 0.40 (0.33-0.49) | 57 958 | 99 | 1.16 (0.94-1.41) | 57 956 | 376 | 1.65 (1.49-1.83) |
| *P_heterogeneity_* |  |  | <.05 |  |  | <.001 |  |  | <.05 |  |  | <.001 |
|  |  |  |  |  |  |  |  |  |  |  |  |  |
| **65-69.9** |  |  |  |  |  |  |  |  |  |  |  |  |
| <20.0 | 26 106 | 16 | 1.77 (1.08-2.90) | 26 122 | 240 | 2.05 (1.81-2.34) | 26 107 | 26 | 0.96 (0.65-1.41) | 26 106 | 46 | 0.65 (0.48-0.86) |
| 20.0-24.9 | 322 792 | 102 | 1.00 (0.82-1.23) | 322 850 | 1253 | 1.00 (0.94-1.06) | 322 797 | 311 | 1.00 (0.89-1.12) | 322 799 | 875 | 1.00 (0.93-1.07) |
| 25.0-29.9 | 283 311 | 114 | 1.15 (0.96-1.38) | 283 340 | 829 | 0.71 (0.67-0.76) | 283 319 | 315 | 1.12 (1.00-1.24) | 283 328 | 1055 | 1.35 (1.27-1.43) |
| 30.0-34.9 | 98 929 | 53 | 1.36 (1.04-1.80) | 98 941 | 232 | 0.53 (0.46-0.60) | 98 932 | 134 | 1.30 (1.09-1.55) | 98 934 | 416 | 1.50 (1.36-1.66) |
| 35+ | 37 785 | 26 | 1.58 (1.06-2.35) | 37 789 | 90 | 0.50 (0.40-0.61) | 37 784 | 50 | 1.21 (0.91-1.61) | 37 784 | 144 | 1.36 (1.15-1.61) |
| *P_heterogeneity_* |  |  | .10 |  |  | <.001 |  |  | .15 |  |  | <.001 |
|  |  |  |  |  |  |  |  |  |  |  |  |  |
| **70+** |  |  |  |  |  |  |  |  |  |  |  |  |
| <20.0 | 13 102 | 20 | 1.60 (1.03-2.49) | 13 114 | 272 | 1.53 (1.36-1.73) | 13 100 | 24 | 1.16 (0.78-1.74) | 13 099 | 42 | 0.83 (0.61-1.13) |
| 20.0-24.9 | 163 770 | 140 | 1.00 (0.84-1.19) | 163 836 | 1915 | 1.00 (0.95-1.05) | 163 762 | 233 | 1.00 (0.87-1.15) | 163 786 | 599 | 1.00 (0.92-1.09) |
| 25.0-29.9 | 152 767 | 135 | 1.01 (0.85-1.19) | 152 824 | 1271 | 0.71 (0.67-0.74) | 152 766 | 234 | 1.04 (0.92-1.18) | 152 778 | 677 | 1.22 (1.14-1.32) |
| 30.0-34.9 | 53 156 | 60 | 1.22 (0.94-1.57) | 53 180 | 418 | 0.65 (0.59-0.72) | 53 160 | 98 | 1.20 (0.98-1.47) | 53 162 | 235 | 1.25 (1.09-1.42) |
| 35+ | 19 017 | 36 | 2.02 (1.43-2.84) | 19 020 | 136 | 0.61 (0.52-0.73) | 19 019 | 42 | 1.40 (1.02-1.92) | 19 019 | 86 | 1.36 (1.09-1.64) |
| *P_heterogeneity_* |  |  | <.05 |  |  | <.001 |  |  | .30 |  |  | <.001 |

BMI indicates body mass index; gsCI, group-specific confidence interval for RR; and RR, relative risk.

^a^ Adjusted for study region, age, socio-economic status, smoking, alcohol consumption, height, heart disease/thrombosis, diabetes mellitus, thyroid disease, rheumatoid arthritis/osteoarthritis, strenuous activity and any activity.

| **Supporting Table 8.** Sensitivity analyses of adjusted^a^ relative risks of forearm (not wrist), wrist and humerus fracture in post-menopausal women according to BMI, stratified by age-bands. | | | | | | | | | |
| --- | --- | --- | --- | --- | --- | --- | --- | --- | --- |
|  | **Forearm (not wrist)** | | | **Wrist** | | | **Humerus** | | |
| **BMI (kg/m^2^)** | **Population at risk** | **Incident cases** | **Adjusted**  **RR (95% gsCI)** | **Population at risk** | **Incident cases** | **Adjusted**  **RR (95% gsCI)** | **Population at risk** | **Incident cases** | **Adjusted**  **RR (95% gsCI)** |
| **50-64.9 years** |  |  |  |  |  |  |  |  |  |
| <20.0 | 40 142 | 83 | 1.40 (1.13-1.75) | 40 144 | 363 | 1.11 (1.00-1.23) | 40 146 | 93 | 1.30 (1.06-1.59) |
| 20.0-24.9 | 476 466 | 681 | 1.00 (0.92-1.08) | 476 468 | 3955 | 1.00 (0.97-1.03) | 476 467 | 784 | 1.00 (0.93-1.08) |
| 25.0-29.9 | 398 181 | 473 | 0.88 (0.81-0.96) | 398 192 | 2612 | 0.82 (0.78-0.85) | 398 184 | 674 | 1.07 (0.99-1.15) |
| 30.0-34.9 | 141 027 | 171 | 0.90 (0.77-1.04) | 141 031 | 735 | 0.65 (0.60-0.70) | 141 028 | 272 | 1.19 (1.05-1.34) |
| 35+ | 55 957 | 80 | 0.98 (0.78-1.23) | 57 956 | 273 | 0.57 (0.51-0.65) | 57 957 | 138 | 1.36 (1.14-1.62) |
| *P_heterogeneity_* |  |  | <.05 |  |  | <.001 |  |  | <.05 |
|  |  |  |  |  |  |  |  |  |  |
| **65-69.9** |  |  |  |  |  |  |  |  |  |
| <20.0 | 26 105 | 45 | 1.49 (1.11-2.01) | 26 119 | 214 | 1.24 (1.08-1.41) | 26 112 | 60 | 1.29 (1.00-1.67) |
| 20.0-24.9 | 322 811 | 360 | 1.00 (0.90-1.11) | 322 844 | 2135 | 1.00 (0.96-1.05) | 322 815 | 535 | 1.00 (0.91-1.09) |
| 25.0-29.9 | 283 314 | 245 | 0.75 (0.66-0.85) | 283 362 | 1529 | 0.79 (0.75-0.83) | 283 341 | 497 | 1.02 (0.94-1.12) |
| 30.0-34.9 | 98 933 | 116 | 0.99 (0.82-1.19) | 98 936 | 446 | 0.65 (0.59-0.71) | 98 933 | 195 | 1.11 (0.97-1.29) |
| 35+ | 37 786 | 25 | 0.56 (0.37-0.83) | 37 788 | 137 | 0.53 (0.45-0.63) | 37 787 | 96 | 1.41 (1.15-1.74) |
| *P_heterogeneity_* |  |  | <.001 |  |  | <.001 |  |  | <.05 |
|  |  |  |  |  |  |  |  |  |  |
| **70+** |  |  |  |  |  |  |  |  |  |
| <20.0 | 13 099 | 33 | 1.42 (1.00-2.00) | 13 109 | 182 | 1.34 (1.15-1.55) | 13 104 | 54 | 0.99 (0.76-1.30) |
| 20.0-24.9 | 163 764 | 280 | 1.00 (0.88-1.13) | 163 816 | 1617 | 1.00 (0.95-1.05) | 163 789 | 614 | 1.00 (0.92-1.09) |
| 25.0-29.9 | 152 765 | 234 | 0.88 (0.77-1.00) | 152 797 | 1184 | 0.77 (0.73-0.82) | 152 788 | 586 | 1.00 (0.93-1.09) |
| 30.0-34.9 | 53 154 | 59 | 0.63 (0.48-0.81) | 53 162 | 327 | 0.62 (0.56-0.69) | 53 164 | 228 | 1.09 (0.95-1.25) |
| 35+ | 19 018 | 27 | 0.82 (0.56-1.21) | 19 022 | 100 | 0.56 (0.46-0.68) | 19 021 | 85 | 1.16 (0.93-1.44) |
| *P_heterogeneity_* |  |  | <.05 |  |  | <.001 |  |  | .67 |

BMI indicates body mass index; gsCI, group-specific confidence interval for RR; and RR, relative risk.

^a^ Adjusted for study region, age, socio-economic status, smoking, alcohol consumption, height, heart disease/thrombosis, diabetes mellitus, thyroid disease, rheumatoid arthritis/osteoarthritis, strenuous activity and any activity.

| **Supporting Table 9.** Sensitivity analyses of adjusted^a^ relative risks femur (not neck), neck of femur, lower leg, and ankle fracture in post-menopausal women according to strenuous activity, stratified by age-bands. | | | | | | | | | | | | |
| --- | --- | --- | --- | --- | --- | --- | --- | --- | --- | --- | --- | --- |
|  | **Femur (not neck)** | | | **Neck of Femur** | | | **Lower Leg** | | | **Ankle** | | |
| **Strenuous activity** | **Population at risk** | **Incident cases** | **Adjusted**  **RR (95% gsCI)** | **Population at risk** | **Incident cases** | **Adjusted**  **RR (95% gsCI)** | **Population at risk** | **Incident cases** | **Adjusted**  **RR (95% gsCI)** | **Population at risk** | **Incident cases** | **Adjusted**  **RR (95% gsCI)** |
|  |  |  |  |  |  |  |  |  |  |  |  |  |
| **50-64.9** |  |  |  |  |  |  |  |  |  |  |  |  |
| Rarely/never (inactive) | 529914 | 276 | 1.00 (0.85-1.18) | 529915 | 1826 | 1.00 (0.94-1.06) | 529918 | 821 | 1.00 (0.91-1.09) | 529920 | 2771 | 1.00 (0.95-1.05) |
| At most once per week | 345709 | 104 | 0.71 (0.59-0.87) | 345711 | 815 | 0.73 (0.68-0.78) | 345710 | 519 | 0.99 (0.91-1.08) | 345713 | 1816 | 1.01 (0.96-1.05) |
| More than once per week | 238149 | 75 | 0.69 (0.54-0.87) | 238153 | 530 | 0.64 (0.59-0.70) | 238153 | 382 | 1.02 (0.92-1.14) | 238152 | 1294 | 1.04 (0.98-1.10) |
| *P_heterogeneity_* |  |  | <.05 |  |  | <.001 |  |  | .91 |  |  | .64 |
|  |  |  |  |  |  |  |  |  |  |  |  |  |
| **65-69.9** |  |  |  |  |  |  |  |  |  |  |  |  |
| Rarely/never (inactive) | 374225 | 199 | 1.00 (0.82-1.21) | 374293 | 1567 | 1.00 (0.94-1.07) | 374228 | 444 | 1.00 (0.89-1.13) | 374243 | 1240 | 1.00 (0.93-1.07) |
| At most once per week | 232448 | 71 | 0.83 (0.65-1.04) | 232477 | 642 | 0.77 (0.71-0.83) | 232460 | 220 | 0.95 (0.83-1.09) | 232457 | 775 | 1.10 (1.02-1.18) |
| More than once per week | 162250 | 41 | 0.66 (0.48-0.90) | 162272 | 435 | 0.70 (0.64-0.78) | 162251 | 172 | 1.02 (0.87-1.20) | 162251 | 521 | 1.06 (0.96-1.16) |
| *P_heterogeneity_* |  |  | .15 |  |  | <.001 |  |  | .77 |  |  | .18 |
|  |  |  |  |  |  |  |  |  |  |  |  |  |
| **70+** |  |  |  |  |  |  |  |  |  |  |  |  |
| Rarely/never (inactive) | 204344 | 258 | 1.00 (0.85-1.17) | 204444 | 2444 | 1.00 (0.95-1.05) | 204339 | 353 | 1.00 (0.87-1.14) | 204356 | 865 | 1.00 (0.92-1.09) |
| At most once per week | 114982 | 71 | 0.63 (0.50-0.80) | 115021 | 927 | 0.83 (0.78-0.89) | 114983 | 162 | 1.03 (0.88-1.20) | 114995 | 456 | 1.04 (0.95-1.14) |
| More than once per week | 82846 | 62 | 0.70 (0.54-0.91) | 82509 | 642 | 0.75 (0.69-0.81) | 82485 | 116 | 1.03 (0.85-1.25) | 82493 | 318 | 0.98 (0.87-1.10) |
| *P_heterogeneity_* |  |  | <.05 |  |  | <.001 |  |  | .96 |  |  | .72 |

gsCI indicates group-specific confidence interval for RR; and RR indicates relative risk.

^a^ Adjusted for study region, age, socio-economic status, smoking, alcohol consumption, height, heart disease/thrombosis, diabetes mellitus, thyroid disease, rheumatoid arthritis/osteoarthritis, BMI and any activity.

| **Supporting Table 10.** Sensitivity analyses of adjusted^a^ relative risks forearm (not wrist), wrist and humerus fracture in post-menopausal women according to strenuous activity, stratified by age-bands. | | | | | | | | | |
| --- | --- | --- | --- | --- | --- | --- | --- | --- | --- |
|  | **Forearm (not wrist)** | | | **Wrist** | | | **Humerus** | | |
| **Strenuous activity** | **Population at risk** | **Incident cases** | **Adjusted**  **RR (95% gsCI)** | **Population at risk** | **Incident cases** | **Adjusted**  **RR (95% gsCI)** | **Population at risk** | **Incident cases** | **Adjusted**  **RR (95% gsCI)** |
|  |  |  |  |  |  |  |  |  |  |
| **50-64.9** |  |  |  |  |  |  |  |  |  |
| Rarely/never (inactive) | 529911 | 700 | 1.00 (0.91-1.10) | 529914 | 3535 | 1.00 (0.96-1.04) | 529918 | 1027 | 1.00 (0.92-1.08) |
| At most once per week | 345709 | 446 | 0.91 (0.83-1.00) | 345715 | 2506 | 1.03 (0.99-1.07) | 345713 | 560 | 0.87 (0.80-0.95) |
| More than once per week | 238153 | 342 | 1.03 (0.91-1.16) | 238162 | 1897 | 1.11 (1.06-1.17) | 238151 | 374 | 0.82 (0.74-0.92) |
| *P_heterogeneity_* |  |  | .22 |  |  | <.05 |  |  | <.05 |
|  |  |  |  |  |  |  |  |  |  |
| **65-69.9** |  |  |  |  |  |  |  |  |  |
| Rarely/never (inactive) | 374237 | 426 | 1.00 (0.89-1.13) | 374292 | 2201 | 1.00 (0.94-1.05) | 374263 | 765 | 1.00 (0.91-1.10) |
| At most once per week | 232459 | 221 | 0.87 (0.76-0.99) | 232484 | 1325 | 0.98 (0.93-1.04) | 232464 | 374 | 0.94 (0.85-1.04) |
| More than once per week | 162253 | 144 | 0.76 (0.64-0.90) | 162273 | 935 | 0.96 (0.89-1.02) | 162261 | 244 | 0.83 (0.73-0.95) |
| *P_heterogeneity_* |  |  | <.05 |  |  | .60 |  |  | .08 |
|  |  |  |  |  |  |  |  |  |  |
| **70+** |  |  |  |  |  |  |  |  |  |
| Rarely/never (inactive) | 204328 | 328 | 1.00 (0.88-1.14) | 204391 | 1805 | 1.00 (0.94-1.06) | 204370 | 896 | 1.00 (0.92-1.09) |
| At most once per week | 114983 | 162 | 0.97 (0.83-1.13) | 115011 | 926 | 0.98 (0.92-1.05) | 115004 | 408 | 0.94 (0.85-1.03) |
| More than once per week | 82849 | 143 | 1.15 (0.92-1.43) | 82504 | 679 | 0.94 (0.87-1.02) | 82492 | 263 | 0.81 (0.72-0.92) |
| *P_heterogeneity_* |  |  | .32 |  |  | .45 |  |  | <.05 |

gsCI indicates group-specific confidence interval for RR; and RR indicates relative risk.

^a^ Adjusted for study region, age, socio-economic status, smoking, alcohol consumption, height, heart disease/thrombosis, diabetes mellitus, thyroid disease, rheumatoid arthritis/osteoarthritis, BMI and any activity.

| **Supporting Table11.** Sensitivity analyses of adjusted^a^ relative risks femur (not neck), neck of femur, lower leg, and ankle fracture in post-menopausal women according to any activity, stratified by age-bands. | | | | | | | | | | | | |
| --- | --- | --- | --- | --- | --- | --- | --- | --- | --- | --- | --- | --- |
|  | **Femur (not neck)** | | | **Neck of Femur** | | | **Lower Leg** | | | **Ankle** | | |
| **Any activity** | **Population at risk** | **Incident cases** | **Adjusted**  **RR (95% gsCI)** | **Population at risk** | **Incident cases** | **Adjusted**  **RR (95% gsCI)** | **Population at risk** | **Incident cases** | **Adjusted**  **RR (95% gsCI)** | **Population at risk** | **Incident cases** | **Adjusted**  **RR (95% gsCI)** |
|  |  |  |  |  |  |  |  |  |  |  |  |  |
| **50-64.9** |  |  |  |  |  |  |  |  |  |  |  |  |
| Rarely/never | 213464 | 130 | 1.00 (0.83-1.20) | 213464 | 790 | 1.00 (0.93-1.08) | 213465 | 351 | 1.00 (0.90-1.11) | 213465 | 1166 | 1.00 (0.94-1.06) |
| At most once per week | 243701 | 83 | 0.63 (0.51-0.78) | 243702 | 633 | 0.74 (0.68-0.80) | 243702 | 356 | 0.90 (0.81-1.00) | 243703 | 1275 | 0.96 (0.91-1.01) |
| 2-3 times week | 238931 | 60 | 0.49 (0.38-0.64) | 238933 | 522 | 0.61 (0.56-0.67) | 238932 | 330 | 0.89 (0.80-0.99) | 238933 | 1187 | 0.97 (0.91-1.03) |
| More than 3 times per week | 311925 | 135 | 0.82 (0.69-0.98) | 311928 | 896 | 0.73 (0.68-0.78) | 311929 | 518 | 1.06 (0.98-1.16) | 311930 | 1700 | 1.10 (1.05-1.16) |
| *P_heterogeneity_* |  |  | <.001 |  |  | <.001 |  |  | <.05 |  |  | <.001 |
|  |  |  |  |  |  |  |  |  |  |  |  |  |
| **65-69.9** |  |  |  |  |  |  |  |  |  |  |  |  |
| Rarely/never | 141815 | 98 | 1.00 (0.81-1.23) | 141840 | 619 | 1.00 (0.92-1.09) | 14817 | 177 | 1.00 (0.86-1.17) | 141820 | 485 | 1.00 (0.91-1.10) |
| At most once per week | 155983 | 51 | 0.58 (0.44-0.76) | 156001 | 488 | 0.83 (0.76-0.90) | 155987 | 156 | 0.91 (0.78-1.07) | 155988 | 501 | 0.99 (0.91-1.08) |
| 2-3 times week | 163739 | 52 | 0.57 (0.43-0.74) | 163759 | 462 | 0.70 (0.64-0.77) | 163742 | 137 | 0.75 (0.63-0.89) | 163743 | 539 | 0.99 (0.91-1.08) |
| More than 3 times per week | 220148 | 73 | 0.57 (0.45-0.71) | 220186 | 748 | 0.76 (0.70-0.81) | 220154 | 253 | 1.00 (0.88-1.14) | 220158 | 735 | 1.01 (0.94-1.09) |
| *P_heterogeneity_* |  |  | <.001 |  |  | <.001 |  |  | <.05 |  |  | .99 |
|  |  |  |  |  |  |  |  |  |  |  |  |  |
| **70+** |  |  |  |  |  |  |  |  |  |  |  |  |
| Rarely/never | 73044 | 103 | 1.00 (0.81-1.23) | 73084 | 913 | 1.00 (0.93-1.07) | 73044 | 142 | 1.00 (0.84-1.19) | 73052 | 306 | 1.00 (0.89-1.12) |
| At most once per week | 74299 | 52 | 0.55 (0.42-0.72) | 74326 | 614 | 0.74 (0.68-0.80) | 74300 | 120 | 0.93 (0.77-1.11) | 74310 | 276 | 0.93 (0.82-1.04) |
| 2-3 times week | 86178 | 63 | 0.56 (0.43-0.71) | 86191 | 724 | 0.69 (0.64-0.75) | 86181 | 102 | 0.67 (0.55-0.81) | 86180 | 341 | 0.95 (0.86-1.06) |
| More than 3 times per week | 118840 | 110 | 0.69 (0.57-0.84) | 118889 | 1170 | 0.75 (0.71-0.80) | 118832 | 190 | 0.88 (0.76-1.02) | 118848 | 493 | 1.00 (0.91-1.09) |
| *P_heterogeneity_* |  |  | <.001 |  |  | <.001 |  |  | <.05 |  |  | .74 |

gsCI indicates group-specific confidence interval for RR; and RR indicates relative risk.

^a^ Adjusted for study region, age, socio-economic status, smoking, alcohol consumption, height, heart disease/thrombosis, diabetes mellitus, thyroid disease, rheumatoid arthritis/osteoarthritis, BMI and strenuous activity.

| **Supporting Table 12.**  Sensitivity analyses of adjusted^a^ relative risks forearm (not wrist), wrist and humerus fracture in post-menopausal women according to any activity, stratified by age-bands. | | | | | | | | | |
| --- | --- | --- | --- | --- | --- | --- | --- | --- | --- |
|  | **Forearm (not wrist)** | | | **Wrist** | | | **Humerus** | | |
| **Any activity** | **Population at risk** | **Incident cases** | **Adjusted**  **RR (95% gsCI)** | **Population at risk** | **Incident cases** | **Adjusted**  **RR (95% gsCI)** | **Population at risk** | **Incident cases** | **Adjusted**  **RR (95% gsCI)** |
|  |  |  |  |  |  |  |  |  |  |
| **50-64.9** |  |  |  |  |  |  |  |  |  |
| Rarely/never | 213461 | 280 | 1.00 (0.89-1.13) | 213462 | 1492 | 1.00 (0.95-1.05) | 213465 | 425 | 1.00 (0.91-1.10) |
| At most once per week | 243702 | 338 | 1.00 (0.90-1.12) | 243703 | 1717 | 0.94 (0.90-0.98) | 243703 | 418 | 0.91 (0.83-1.01) |
| 2-3 times week | 238933 | 289 | 0.89 (0.79-0.99) | 238936 | 1725 | 0.95 (0.91-1.00) | 238933 | 370 | 0.86 (0.78-0.95) |
| More than 3 times per week | 311925 | 430 | 1.00 (0.91-1.10) | 311938 | 2331 | 0.99 (0.95-1.03) | 311929 | 553 | 0.96 (0.88-1.04) |
| *P_heterogeneity_* |  |  | .33 |  |  | .22 |  |  | .19 |
|  |  |  |  |  |  |  |  |  |  |
| **65-69.9** |  |  |  |  |  |  |  |  |  |
| Rarely/never | 141827 | 161 | 1.00 (0.85-1.17) | 141841 | 801 | 1.00 (0.93-1.07) | 141823 | 317 | 1.00 (0.89-1.12) |
| At most once per week | 155984 | 146 | 0.84 (0.71-0.99) | 156007 | 851 | 0.99 (0.92-1.06) | 155996 | 255 | 0.83 (0.73-0.93) |
| 2-3 times week | 163749 | 152 | 0.77 (0.66-0.91) | 163764 | 972 | 0.98 (0.92-1.05) | 163752 | 250 | 0.75 (0.66-0.85) |
| More than 3 times per week | 220156 | 243 | 0.88 (0.77-1.00) | 220186 | 1344 | 0.96 (0.91-1.02) | 220168 | 409 | 0.88 (0.80-0.97) |
| *P_heterogeneity_* |  |  | .16 |  |  | .86 |  |  | <.05 |
|  |  |  |  |  |  |  |  |  |  |
| **70+** |  |  |  |  |  |  |  |  |  |
| Rarely/never | 73036 | 110 | 1.00 (0.82-1.21) | 73060 | 634 | 1.00 (0.92-1.08) | 73052 | 301 | 1.00 (0.89-1.13) |
| At most once per week | 74298 | 102 | 0.95 (0.78-1.15) | 74318 | 535 | 0.84 (0.77-0.92) | 74315 | 269 | 0.99 (0.88-1.12) |
| 2-3 times week | 86184 | 130 | 0.97 (0.82-1.15) | 86201 | 710 | 0.88 (0.82-0.95) | 86190 | 301 | 0.92 (0.82-1.03) |
| More than 3 times per week | 118831 | 183 | 0.95 (0.82-1.10) | 118865 | 1094 | 0.95 (0.89-1.00) | 118853 | 454 | 0.96 (0.87-1.05) |
| *P_heterogeneity_* |  |  | .23 |  |  | <.05 |  |  | .73 |

gsCI indicates group-specific confidence interval for RR; and RR indicates relative risk.

^a^ Adjusted for study region, age, socio-economic status, smoking, alcohol consumption, height, heart disease/thrombosis, diabetes mellitus, thyroid disease, rheumatoid arthritis/osteoarthritis, BMI and strenuous activity.
